# Poly-*β*-hydroxybutyrate Production from Bread Waste via Sequential Dark Fermentation and Photofermentation

**DOI:** 10.3390/foods14101659

**Published:** 2025-05-08

**Authors:** Luca Bernabò, Giulia Daly, Gianmarco Mugnai, Viola Galli, Elisa Corneli, Lisa Granchi, Alessandra Adessi

**Affiliations:** 1 Department of Agriculture, Food, Environment and Forestry (DAGRI), University of Florence, Piazzale delle Cascine, 18, 50144 Florence, Italy; luca.bernabo@unifi.it (L.B.); giulia.daly@unifi.it (G.D.); gianmarco.mugnai@unipd.it (G.M.); cornelielisa@gmail.com (E.C.); lisa.granchi@unifi.it (L.G.); 2 Department of Agronomy, Food, Natural Resources, Animals and Environment, Viale dell’Università 16, 35020 Legnaro, Italy; 3 FoodMicroTeam s.r.l., Academic Spin-Off of University of Florence, Via Santo Spirito 14, 50125 Florence, Italy

**Keywords:** lactic acid fermentation, *Cereibacter johrii* Pisa7, fermented bread broth, LED-optimized photobioreactor, purple non-sulfur bacteria

## Abstract

This study explores the valorization of bread waste for poly-*β*-hydroxybutyrate (PHB) production through a combined dark fermentation (DF) and photofermentation (PF) process. DF, performed using *Lactobacillus amylovorus* DSM 20532, efficiently converted bread waste into a lactate- and acetate-rich substrate within 120 h. The resulting fermented bread broth (FBB) was enriched with essential nutrients by adding digestate from anaerobic digestion, replacing the need for chemical supplements. Six purple non-sulfur bacteria (PNSB) strains were screened for PHB production in the FBB. *Cereibacter johrii* Pisa7 demonstrated the highest PHB accumulation (50.73% w PHB/w cells), and biomass increase (+1.26 g L^−1^) over 336 h, leading to its selection for scale-up. Scale-up experiments were conducted in a 5 L photobioreactor with LED lights optimized for PNSB growth. *C. johrii* Pisa7 accumulated PHB at 15.17% and 11.51% w PHB/w cells in two independent trials, corresponding to productivities of 2.03 and 0.89 mg PHB L^−1^ h^−1^. These results confirm the scalability of the process while maintaining competitive PHB yields. This study highlights the potential of bread waste as a low-cost carbon source for bioplastic production, contributing to a circular bioeconomy by converting food waste into sustainable materials.

## 1. Introduction

Since the 1950s, plastic consumption has increased significantly, driven by demographic and economic growth and favored by its unique properties, including lightness, durability, versatility, and low production costs [1,2]. However, the widespread use of petroleum-based plastics has led to severe environmental issues due to their persistence and disposal challenges, particularly in low-income countries [3,4]. Growing awareness of their ecological impact and the need to reduce reliance on fossil fuels has accelerated the search for sustainable alternatives.

In this context, bioplastics derived from renewable biomass, such as biopolymers, are emerging as innovative materials that offer properties comparable to conventional plastics but with a lower environmental footprint [5,6].

Among biopolymers, polyhydroxyalkanoates (PHAs) have garnered increasing interest. They are valued for their biodegradability, biocompatibility, water insolubility, UV resistance, and thermoplastic properties, making them promising substitutes for conventional plastics [7].

PHAs are polyesters synthesized by bacteria, archaea, and yeasts as energy reserves, especially under nutrient-deficient conditions (e.g., high C/N ratios or limited phosphorus, sulfur, or magnesium) [8,9,10,11,12].

Currently, most biopolymers are produced from first- and second-generation feedstocks. First-generation sources, such as maize and sugar beet, compete with food and feed production, requiring arable land, fertilizers, and water, thus contributing to environmental impacts. Second-generation feedstocks, including lignocellulosic biomass and organic waste, mitigate this competition but require costly chemical and enzymatic treatments for processing [13]. To address these limitations, third-generation feedstocks, such as microbes, are gaining attention as a more sustainable alternative. They do not compete for arable land or freshwater resources and facilitate biomass conversion into biopolymers, reducing costs and environmental impact [13].

Poly-*β*-hydroxybutyrate (PHB), a short-chain-length PHA (Scl-PHA) that is the most extensively studied member of this family [14,15], is synthesized by various microorganisms, including photosynthetic bacteria, using simple feedstocks such as carbohydrates, organic acids, alcohols, and glycerol [14].

Purple non-sulfur bacteria (PNSB) have the capability to accumulate PHB [7,16,17,18]. Under anaerobic conditions, PNSB can perform photofermentation (PF), converting organic acids into PHB while utilizing light as an energy source [7,19]. These bacteria can also synthesize biohydrogen (bio-H_2_) via nitrogenase enzyme, although PHB biosynthesis competes with bio-H_2_ production for reductants [20]. The most studied PNSB species for PHB production include *Cereibacter sphaeroides* (formerly *Rhodobacter shpaeroides*), *Rhodopseudomonas palustris*, *Rhodobacter capsulatus*, *Rhodobacter sulfidophilus*, and *Rhodospirillum rubrum* [16,21,22].

Despite its potential, the industrial-scale production of PHAs faces economic barriers, with costs of USD 3–5/kg compared to USD 1–1.50/kg for conventional plastics [23]. A significant portion (30–50%) of PHA production costs stem from the carbon source used in microbial growth media. To address this, utilizing low-cost, abundant carbon sources derived from food waste, combined with metabolically versatile microorganisms, offers a promising strategy to reduce costs [19,24,25].

Integrating PF with dark fermentation (DF) offers an efficient way to utilize food waste for PHA production. During DF, heterotrophic bacteria first convert sugars contained in food waste into organic acids, which PNSB subsequently transform into PHB and/or bio-H_2_ during PF.

Bread waste, which constitutes a significant contributor to household food waste in Europe, is a promising substrate for microbial fermentation due to its high carbohydrate content, primarily in the form of starch (~70%), [26]. In the European Union, 88 million tons of food waste are generated annually, with bread comprising approximately 19% of household waste in Italy [26,27]. The utilization of bread waste in fermentation can lead to the production of value-added products, thereby enhancing resource efficiency and contributing to circular economy initiatives. Therefore, amylolytic lactic acid bacteria (LAB) can efficiently convert starch-rich bread waste into lactate through DF, avoiding the need for added amylolytic enzymes. One of these amylolytic LAB is *Lactobacillus amylovorus*, which has been shown to produce lactate from starch-based substrates [26,28,29,30,31,32]. The lactate produced can then be converted into PHB or bio-H_2_ through PNSB-driven PF.

Despite the promising potential of bread waste as a substrate for PHB production, comprehensive research in this area remains limited. Critical factors influencing the fermentation process (such as the impact of light quality on PNSB) have not been extensively explored. Since the efficiency of PHB and bio-H_2_ production in PNSB is highly dependent on light quality, with bacteriochlorophylls (BChls) absorbing light at 590 nm and in the near-infrared range (800–880 nm) [33], the incident light spectrum can be designed to enhance PF.

The present study aimed to evaluate six PNSB strains for their ability to grow and produce PHB using bread waste as a substrate. A combined system was implemented, consisting of an initial DF stage to enrich the waste stream with organic acids, followed by a PF stage, minimizing the need for extensive pretreatment of the waste stream. The PNSB strain demonstrating the highest growth and PHB production was then selected for scale-up in a 5 L photobioreactor equipped with LED lights emitting in the yellow (λ = 593 nm) and infrared (λ = 860 nm) spectra.

## 2. Materials and Methods

### 2.1. Bacterial Strains

*L. amylovorus* DSM 20532 was obtained from the German Collection of Microorganisms and Cell Cultures GmbH, Leibniz Institute DSMZ, Braunschweig, Germany [34].

Six previously identified PNSB strains [35] were selected to assess their capability to produce PHB via PF using bread waste. Salient information on the PNSB strains used in this study is listed in Table 1. The microorganisms were stored in sealed agar tubes at the Department of Agricultural, Food, Environment and Forestry (DAGRI) of the University of Florence, Italy.

### 2.2. Inoculum and Dark Fermentation Assay Using Bread Waste

*L. amylovorus* DSM 20532 was cultured for 24 h in MR3i medium containing peptone 5 g L^−1^; tryptone 5 g L^−1^; lab-lemco 5 g L^−1^; yeast extract 12 g L^−1^; maltose 20 mg L^−1^; glucose 6 g L^−1^; fructose 6 g L^−1^; sodium gluconate 2 g L^−1^; sodium acetate 2 g L^−1^; ammonium citrate 2 g L^−1^; dipotassium hydrogen phosphate 2 g L^−1^; magnesium sulfate 7·H_2_O 0.2 g L^−1^; manganese sulfate 10·H_2_O 0.05 g L^−1^; cysteine 0.5 g L^−1^; vitamin mix 1000 × 1 mL L^−1^; Tween 80 1 mL L^−1^; fresh yeast extract 15 mL L^−1^; and distilled water. The pH of the medium was adjusted to 5.6. Stationary phase cells were collected by centrifugation at 1500× *g* for 15 min (min) at 22 °C using a Sigma centrifuge model 3–16KL (Sigma Laborzentrifugen GmbH, Osterode am Harz, Germany) and resuspended in bread effluent for the DF phase.

To obtain the fermented bread broth (FBB) for the PF tests, bread waste was oven-dried overnight at 60 °C and then ground with a mixer to a particle size of 500–1000 µm. *L. amylovorus* DSM 20532 was inoculated at a final concentration of 1 × 10^9^ CFU mL^−1^ into a substrate consisting of 7.5% bread/water (*w***/***v*).

To optimize the duration of the DF process, triplicate fermentation tests were conducted using the inoculated bread substrate, in 100 mL Erlenmeyer flasks. The flasks were incubated at 37 °C and subjected to horizontal shaking at 100 rpm for 168 h (h). Throughout the experiment, the concentrations of organic acids were monitored at 24 h intervals using high-performance liquid chromatography (HPLC) analysis to track their variations over time. Additionally, pH changes in the substrate were recorded every 24 h.

The DF to produce FBB for subsequent PF tests was carried out in 3 L Erlenmeyer flasks under the same conditions described above and lasted for 120 h. The FBB was obtained by filtering the dark fermented bread waste with a metal sieve, followed by centrifugation at 3800× *g* rpm for 15 min at 22 °C using a Beckman centrifuge model J2-21 (Beckman Coulter, Brea, CA, USA). The resulting effluent was supplemented with: (1) 10 mL L^−1^ of digestate from an anaerobic digestion (AD) plant of Società Agricola Lomas s.r.l., Collesalvetti (LI), Italy, and (2) 10 mL L^−1^ of a phosphate buffer (K_2_HPO_4_, 50 g L^−1^; KH_2_PO_4_, 30 g L^−1^). The digestate had been pre-filtered using 1.2 μm mixed cellulose ester (MCE) membranes (Fisher Scientific International, Inc., Pittsburgh, PA, USA). Finally, the FBB was sterilized at 121 °C for 15 min using a Vapormatic autoclave model 770 (Carlo Erba Reagents s.r.l., Cornaredo, MI, Italy) to prevent residual lactic acid bacteria metabolic activity, which could lower the pH, and to maintain it within the optimal range (6.5–7.0) for PHB production [7]. The pH of the substrate was adjusted to 6.8 before and after autoclaving.

### 2.3. Inoculum and Batch Photofermentation Assays Using Fermented Bread Broth

Preliminary batch PF screening tests were conducted using pure cultures of each PNSB strain to identify the most effective PHB-producing candidate. Before the experiments, the PNSB strains were activated in 250 mL bottles containing RPN medium supplemented with DL-lactic acid (1.8 g L^−1^) as the carbon source. Cultures were incubated at 25 °C under continuous illumination at 150 μmol photons m^−2^ s^−1^.

The RPN medium consisted of NH_4_Cl 0.5 g L^−1^, K_2_HPO_4_ 0.5 g L^−1^, KH_2_PO_4_ 0.3 g L^−1^, MgSO_4_·7H_2_O 0.4 g L^−1^, NaCl 0.4 g L^−1^, CaCl_2_·2H_2_O 0.075 g L^−1^, ferric citrate 0.005 g L^−1^, and yeast extract 0.4 g L^−1^. Trace elements were added at 10 mL per liter from a stock solution containing ZnSO_4_·7H_2_O 10 mg L^−1^, MnCl_2_·4H_2_O 3 mg L^−1^, H_3_BO_3_ 30 mg L^−1^, CoCl_2_·6H_2_O 20 mg L^−1^, CuCl_2_·2H_2_O 1 mg L^−1^, NiCl_2_·6H_2_O 2 mg L^−1^, and Na_2_MoO_4_·2H_2_O 30 mg L^−1^. The medium pH was adjusted to 6.8 using NaOH prior to autoclaving.

For the PF batch tests, each PNSB strain was harvested by centrifugation at 3000× *g* for 20 min at 22 °C using a Beckman J2-21 centrifuge (Beckman Coulter, Brea, CA, USA). The cell pellets were then resuspended in FBB, which served as the test substrate. Each culture was inoculated to reach a final biomass concentration of 100 mg L^−1^.

PF assays were carried out in sealed 100 mL serum bottles equipped with rubber stoppers and clamps. To allow biohydrogen (bio-H_2_) release while preventing microbial contamination, each bottle was fitted with a needle equipped with a 0.2 µm syringe filter (Lab Logistics Group GmbH, Meckenheim, Germany).

Before the PF trials, the FBB medium was analyzed for macro- and micronutrients, organic acids, and ammonium content. The bottles were incubated under batch conditions at 25 °C with continuous white LED light (150 μmol photons m^−2^ s^−1^). All experiments were performed in triplicate and lasted 336 h.

Cell dry weight (CDW) was measured at both the beginning (T0, 0 h) and the end (T14, 336 h) of the experiment. PHB accumulation and transmission electron microscopy (TEM) observations were carried out at the end of the test (T14) to assess the presence of intracellular PHB granules. Further details on the analytical procedures are provided in Section 2.5 (Analytical Methods).

### 2.4. Photobioreactor Design and Scale-Up of PHB Production

The scale-up PF tests were set up in a tubular photobioreactor designed by Biosyntex s.r.l. (Bologna, BO, Italy) and constructed by Tecnocom s.r.l. (Prato, PO, Italy). The system consisted of a skid of dimensions 515 mm × 400 mm, comprising (1) a thermostated DURAN glass tube, measuring H 700 mm × D 140 mm and equipped with an internal chamber of D 110 mm; (2) a pH control instrument (B&C Electronics s.r.l., Carnate, MB, Italy); (3) a temperature control instrument (Gefran S.p.a., Provaglio d’Iseo, BS, Italy); (4) a mechanical stirrer model AM20-D 50 (Argolab, Carpi, MO, Italy) with manual speed control and a polypropylene (PP) rod (H 800 mm × D 8 mm); (5) two 150W dimmable LED lamps, measuring 600 × 70 × 40 mm (Ambra Elettronica s.r.l., Bolzano Vicentino, VI, Italy); (6) an ON/OFF electric valve for thermostatic control; (7) an electrical control and command panel. Each LED lamp consists of 32 LEDs with an emission spectrum in the yellow region (λ = 593 nm) and 8 LEDs with an emission spectrum in the infrared region (λ = 860 nm).

The 5 L photobioreactor was used for the scale-up of the PHB production process, employing *Cereibacter johrii* Pisa7, the strain selected during the preliminary PF screening. Before inoculation, the strain was pre-activated in an RPN medium supplemented with DL-lactic acid, as described in Section 2.3 (Inoculum and Batch Photofermentation Assays using Fermented Bread Broth). The culture was then harvested by centrifugation and resuspended in fermented bread broth (FBB), following the same procedure outlined in Section 2.3. Inoculation into the photobioreactor was performed to reach a final optical density at 660 nm (OD_660_) of 0.2. Before inoculation, the substrate was reanalyzed to determine its organic acid and ammonium content. The experiment was conducted twice as independent trials (named Run_1 and Run_2).

Before each experiment, the 5 L photobioreactor was disinfected through the following steps: (1) initial cleaning and rinsing of the column with non-sterile deionized water; (2) filling of the column with non-sterile deionized water and addition of 0.5 mL L^−1^ of a commercial 15% NaClO solution (150 g L^−1^ of active chlorine), followed by continuous stirring at 60 rpm for 12 h; (3) addition of 1 ppm Na_2_S_2_O_3_·5H_2_O for each ppm of active chlorine and continuous stirring at 60 rpm for 30 min; (4) double rinsing with sterile deionized water.

The scale-up experiments lasted a total of 336 h. Each day, the culture was kept static except for a 30 min period during which it was agitated at 170 rpm. Microbial growth was monitored by quantifying bacteriochlorophyll *a* (BChl*a*) every 48 h and by determining CDW at T0 (0 h), T7 (168 h), and T14 (336 h). Organic compounds and ammonium concentrations were assessed at T0, T7, and T14 h using HPLC and the Nessler method, respectively. PHB content was measured at T0, T7, and T14. Further details on the analytical methods used are provided in Section 2.5 (Analytical Methods).

Throughout the experiment, light intensity was increased stepwise. During the first 24 h, it was set at 150 μmol photons m^−2^ s^−1^. Between 24 and 120 h, it was raised to 250 μmol photons m^−2^ s^−1^, and from 120 h onward, it was stabilized at 400 μmol photons m^−2^ s^−1^ until the end of the experiment.

### 2.5. Analytical Methods

#### 2.5.1. FBB Chemical Characterization

The concentration of metals and other inorganic compounds of the FBB was determined through an inductively coupled plasma–optical emission spectrometry ICP-OES analyzer (Thermo Scientific™ iCAP™ 7400, Waltham, MA, USA), using IRSA 3010 (conventional acid mineralization) and IRSA A-3020 (determination of chemical elements by spectroscopy emission with plasma source) methods.

The organic compounds content of the FBB was determined using a Varian Pro Star HPLC system equipped with an Aminex 87H column maintained at 65 °C. The system utilized a 20 µL injection loop, a UV detector (λ = 210 nm), and a refractive index (RI) detector. The eluent was 0.01 N H_2_SO_4_, at a flow rate of 0.6 mL min^−1^.

The ammonium concentration was determined using the HI93764B-25 Ammonia HR reagents set (Hanna Instruments, Woonsocket, RI, USA), based on the Nessler method, and quantified spectrophotometrically using a Varian Cary50 UV-visible spectrophotometer (Varian, Mulgrave, Australia).

#### 2.5.2. C/N Ratio and Organic Acids Yield Calculation

The C/N ratio of the substrates was calculated by dividing the sum of the moles of carbon contained in lactate and acetate by the moles of nitrogen in ammonium.

The fermentation efficiency in terms of organic acid yield was calculated as the ratio between the sum of lactic and acetic acid concentrations (g L^−1^), produced during dark fermentation, and the total carbohydrate content present in the diluted substrate (7.5% *w*/*v*). The carbohydrate content per 100 g of bread was derived from Adessi et al. [26], as the same substrate was used in the present study.

#### 2.5.3. Monitoring of PNSB Growth

CDW was determined in triplicate on a 3 mL culture sample, through a filtration with 0.45 μm mixed cellulose ester (MCE) membranes (Fisher Scientific International, Inc., Pittsburgh, PA, USA). The membranes had been previously activated with 5 mL of distilled water. After filtration, the cells were washed twice with distilled water, oven-dried at 105 °C for 3 h, and weighed on an analytical balance (Sartorius AG, Göttingen, Germany).

BChl*a* content was determined according to Carlozzi and Sacchi [39]. Briefly, a 2 mL sample was centrifugated at 4000× *g* for 10 min with a Hermle Z 167 M centrifuge (Hermle Labortechnik GmbH, Wehingen, Germany), and the supernatant was removed. BChl*a* was extracted from the cell pellet, resuspending it with a 2 mL acetone/methanol 7:2 *v*/*v* ratio solution. The sample was incubated at 4 °C for 30 min and centrifugated at 4000× *g* for 10 min. The BChl*a* content was determined by measuring the absorbance (A) at 775 nm (ε = 75 mM^−1^ cm^−1^) with a Varian Cary50 UV-visible spectrophotometer (Varian, Mulgrave, Australia).

The optical density was determined in triplicate on 2 mL samples at a wavelength of 660 nm (OD_660_) using a Varian Cary50 UV-visible spectrophotometer (Varian, Mulgrave, Australia). The RPN medium was used as a blank.

#### 2.5.4. Transmission Electron Microscopy (TEM) Analysis

TEM observations were conducted at the end of the preliminary screening test (336 h) on the FBB substrate, using a Philips CM12 TEM (Philips Electronics, Amsterdam, The Netherlands) equipped with CRYO-GATAN UHRST 3500 technology and a digital camera. Before image acquisition, bacterial cells were prepared as follows: the cells were treated with 2.5% (*w*/*v*) glutaraldehyde at 4 °C and then washed twice in 0.1 M phosphate buffer (PBS), pH 7.0, for 15 min each. Subsequently, the cells were fixed with 1% osmium tetroxide, pH 7, for 1 h. The samples were then suspended in PBS and air-dried. Next, the samples were gradually rehydrated in an ethanol series (25%, 50%, 70%, 80%, 90%, and 100%) for 10 min at each concentration. Spurr’s epoxy resin was used for infiltration and embedding of the samples. The epoxy resin was first incubated in propylene oxide for 10 min in a 2:1 mixture of propylene oxide/resin, followed by a 1:1 mixture for at least 1 h, and finally in a 1:2 mixture of propylene oxide/resin for 1 h to overnight. The samples were cut and post-stained with uranyl acetate and lead citrate.

#### 2.5.5. PHB Quantification

PHB production was measured following the method described by De Philippis et al. [40] through acid cellular hydrolysis. It was quantified as crotonic acid using a Varian Pro Star HPLC system, equipped with an Aminex 87H column maintained at 65 °C. The system employed a 20 μL injection loop, a UV detector (λ = 210 nm), and a refractive index (RI) detector. The eluent was 0.01 N H_2_SO_4_ (Carlo Erba, Milan, Italy) at a flow rate of 0.6 mL min^−1^.

### 2.6. Statistical Analysis

All the analyses were conducted in experimental triplicates (*N* = 3); a minimum number of three instrumental replicates was always used for each measurement (*n* = 3).

To determine whether the results were significantly different, data were analyzed using independent *t*-tests and one-way analysis of variance (ANOVA) at 95% of the significance, followed by Tukey’s honest significance difference (HSD) post hoc test. Results were considered statistically significant at *p* < 0.05. Before the t-test, all data were checked for normality using the Shapiro–Wilk test. Before ANOVA, normality was assessed with the Shapiro–Wilk test, and homogeneity of variance was evaluated using Bartlett’s test. Statistical analysis was performed using R software version 4.4.1.

## 3. Results

### 3.1. Dark Fermentation (DF) Optimization

**Figure 1:**
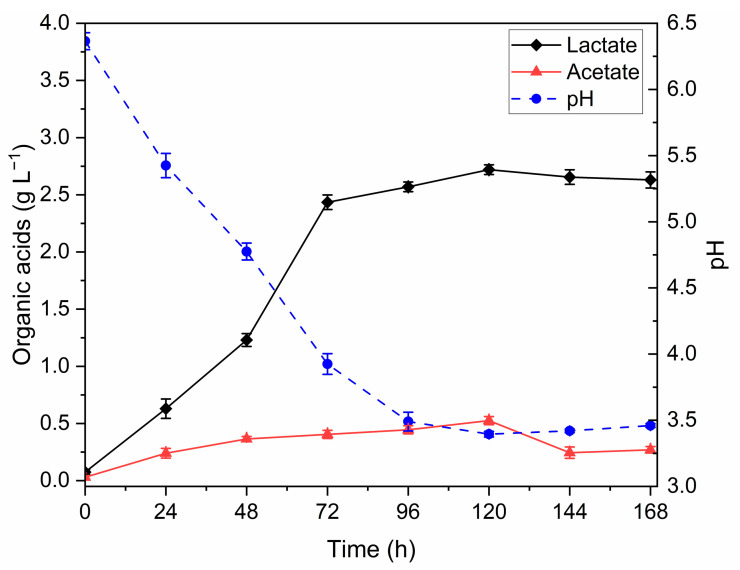
Time-course profiles of lactate, acetate, and pH during the optimization process of DF.

The DF process of bread waste using *L. amylovorus* DSM 20532 was optimized to determine the optimal fermentation duration and maximize lactate yield, as shown in Figure 1. The highest lactate concentration was recorded after 120 h, reaching 2.72 (±0.04) g L^−1^. Acetate production followed a similar trend, peaking at 0.53 (±0.04) g L^−1^ at the same time point. This pattern was further supported by the pH values, which reflect the accumulation of these organic acids. Based on these findings, the optimal fermentation time was established at 120 h, and all subsequent bread waste fermentations were conducted for this duration.

### 3.2. FBB Chemical Composition

The metal and inorganic element composition of the FBB, after the digestate addition, used in the PF experiments is reported in Table 2.

The lactate, acetate, and ammonium content, the C/N ratio, and the organic acids yield in the FBB substrates used for the three PF trials are reported in Table 3. One-way ANOVA revealed no statistically significant differences among the substrates for lactate, acetate, ammonium, the C/N ratio, and the organic acids yield.

### 3.3. Screening of PNSB Strains for Growth and PHB Production on FBB

**Figure 2:**
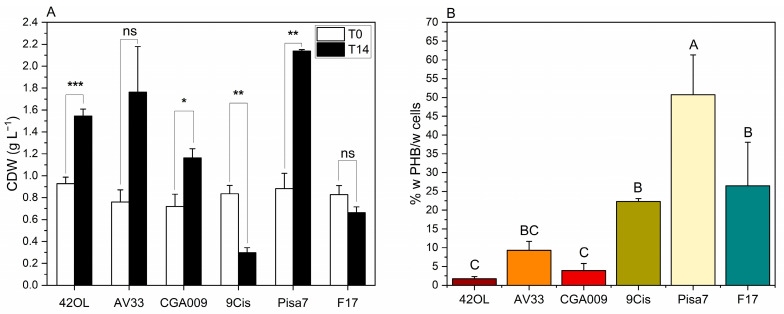
Growth and PHB production in six PNSB strains (*R. palustris* 42OL, *R. palustris* AV33, *R. palustris* CGA009, *C. johrii* 9Cis, *C. johrii* Pisa7, and *C. sphaeroides* F17) during the preliminary screening test. (**A**) CDW (g L^−1^) at the start (T0) and end (T14) of the trial. Statistical significance between time points was assessed for each strain using a paired *t*-test, with significant differences indicated by asterisks (* *p* < 0.05, ** *p* < 0.01, *** *p* < 0.001) and “ns” for not significant. Error bars represent standard deviations (*n* = 3). (**B**) PHB content (w PHB/w cells) measured at T14. Differences among strains were analyzed using one-way ANOVA, followed by Tukey’s HSD post hoc test, with significance indicated by uppercase letters (A, B, C). Error bars represent standard deviations (*n* = 3).

The first PF test aimed to assess the growth and PHB synthesis capabilities of six different PNSB strains cultivated on FBB by using batch setups. Figure 2 illustrates the variation in CDW over time and the PHB accumulation measured at the end of the trial.

In Figure 2A, cellular growth, expressed as CDW (g L^−1^), is compared between the initial time point (T0, white bars) and after 14 days of cultivation (T14, black bars). A paired *t*-test revealed significant differences (*p* < 0.05, *) in four out of six strains. Notably, *R. palustris* 42OL, *R. palustris* CGA009, and *C. johrii* Pisa7 exhibited a significant increase in CDW over the cultivation period, with *C. johrii* Pisa7 showing the highest increase (+1.26 g L^−1^ ± 0.15). In contrast, *C. johrii* 9Cmis displayed a marked decrease in CDW (−0.54 g L^−1^ ± 0.10). Meanwhile, *R. palustris* AV33 and *C. sphaeroides* F17 showed no significant variation in CDW over the tested period.

Figure 2B illustrates the accumulation of PHB (% w PHB/w cells) in the six tested strains, quantified at the end of the experiment (T14). *C. johrii* Pisa7 significantly exhibited the highest PHB accumulation (50.73% w PHB/w cells ± 10.60), followed by *C. sphaeroides* F17 (26.47% w PHB/w cells ± 11.58) and *C. johrii* 9Cis (22.31% w PHB/w cells ± 0.77). In contrast, *R. palustris* strains (42OL, AV33, and CGA009) displayed no significant differences in PHB accumulation levels, which were below 10% w PHB/w cells.

The volumetric production of PHB at T14, together with hourly and daily productivity, is presented in Table 4. *C. johrii* Pisa7 showed the highest volumetric production of PHB, reaching 1083.76 ± 220.05 mg PHB L^−1^, with a daily productivity of 77.41 ± 15.72 mg PHB L^−1^ d^−1^. This was followed by *C. sphaeroides* F17 (179.47 ± 92.29 mg PHB L^−1^, 12.82 ± 6.59 mg PHB L^−1^ d^−1^) and *R. palustris* AV33 (159.68 ± 42.02 mg PHB L^−1^, 11.41 ± 3.00 mg PHB L^−1^ d^−1^). The remaining strains showed significantly lower PHB production, with values below 100 mg PHB L^−1^ and daily productions below 10 mg PHB L^−1^ d^−1^.

**Figure 3:**
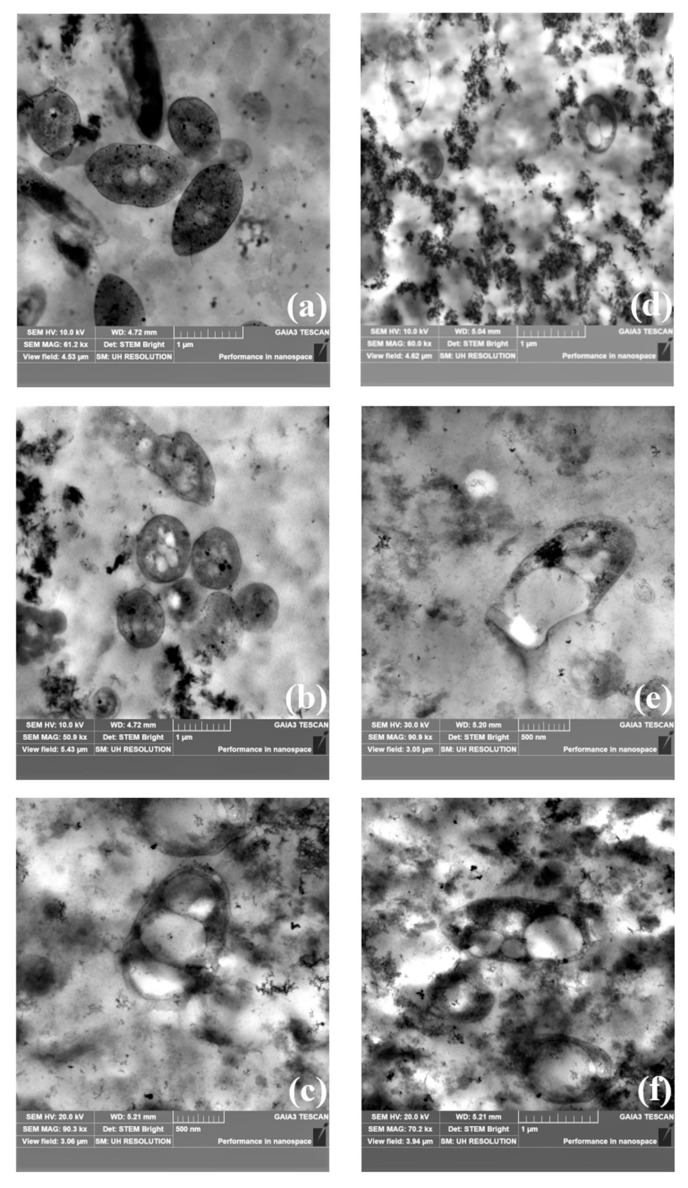
Transmission electron microscopy (TEM) images of the inclusion bodies from screening tests conducted using *R. palustris* strain 42OL (**a**), *R. palustris* strain AV33 (**b**), *R. palustris* strain CGA009 (**c**), *C. johrii* strain Pisa7 (**d**), *C. johrii* strain 9Cis 7 (**e**), and *C. sphaeroides* strain F17 (**f**) growth on FBB at the end of the photofermentation experiment (T14).

At the end of the screening test (T14), TEM observations confirmed the presence of cytoplasmic PHB inclusions in all the analyzed samples (Figure 3). Among the *R. palustris* strains, *R. palustris* 42OL (Figure 3a) exhibited the largest cell length (L), with an average of 1.42 ± 0.11 µm, followed by *R. palustris* CGA009 (1.34 ± 0.08 µm) and *R. palustris* AV33 (1.14 ± 0.09 µm), respectively, in Figure 3b and Figure 3c. In terms of cell width (W), *R. palustris* 42OL also ranked highest, averaging 0.84 ± 0.04 µm, slightly exceeding *R. palustris* CGA009 (0.82 ± 0.03 µm) and *R. palustris* AV33 (0.80 ± 0.07 µm). Regarding PHB inclusions, *R. palustris* CGA009 stood out with the largest inclusion dimensions, displaying an average W of 0.49 ± 0.17 µm and L of 0.65 ± 0.18 µm, compared to *R. palustris* 42OL (W: 0.22 ± 0.04 µm; L: 0.21 ± 0.04 µm) and *R. palustris* AV33 (W: 0.17 ± 0.01 µm; L: 0.25 ± 0.02 µm).

For the *Cereibacter* genus, *C. sphaeroides* F17 (Figure 3f) displayed the largest cell dimensions, with an average length (L) of 1.73 ± 0.24 µm and width (W) of 0.81 ± 0.07 µm, followed by *C. johrii* 9Cis (L: 1.16 ± 0.37 µm, W: 0.78 ± 0.04 µm) and *C. johrii* Pisa7 (L: 0.86 ± 0.01 µm, W: 0.56 ± 0.04 µm), respectively, in Figure 3d and Figure 3e. Similarly, PHB inclusion size reflected these trends. *C. sphaeroides* F17 showed the largest inclusions, with an average W of 0.50 ± 0.05 µm and L of 0.61 ± 0.10 µm. *C. johrii* 9Cis followed with inclusion dimensions of 0.48 ± 0.12 µm (W) and 0.59 ± 0.12 µm (L), while *C. johrii* Pisa7 displayed the smallest inclusions, with a W of 0.17 ± 0.05 µm and L of 0.30 ± 0.11 µm.

### 3.4. Scale-Up of PHB Production in a 5 L Photobioreactor

Based on the results presented in Section 3.3 (Screening of PNSB Strains for Growth and PHB Production on FBB), *C. johrii* Pisa7 was selected for the subsequent scale-up of the process in a 5 L photobioreactor. The photobioreactor was equipped with LED lamps emitting light at two specific wavelengths, 593 nm and 860 nm. *C. johrii* Pisa7 was cultivated on FBB for 14 days (336 h), and the experiment was conducted twice as independent trials. These trials are referred to as Run_1 and Run_2 in the following text.

**Figure 4:**
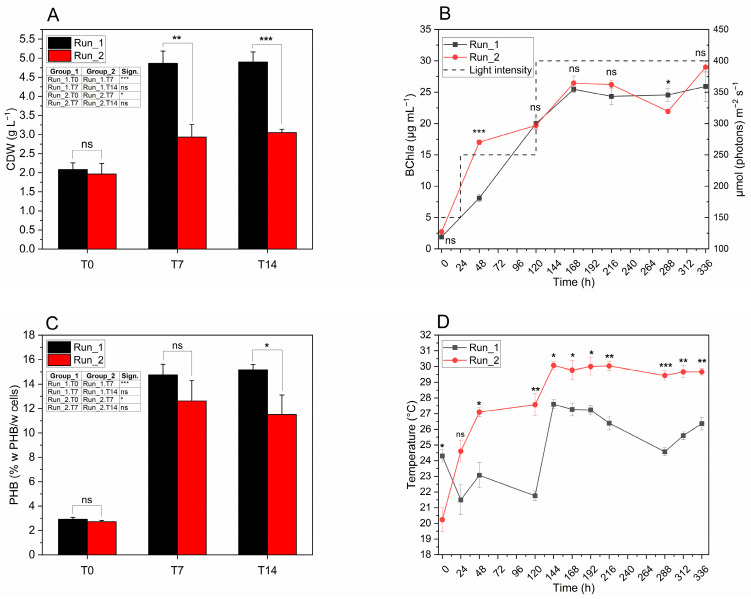
Profiles of CDW, BChl*a*, PHB, and temperature during the experiments. (**A**) Cell dry weight (CDW, g L^−1^) at T0 (0 h), T7 (168 h), and T14 (336 h). (**B**) Time course of BChl*a* synthesis. (**C**) PHB content (% w PHB/w Cells) at T0, T7, and T14. (**D**) Temperature profile (°C). Statistical differences between runs at the same time point were assessed using independent *t*-tests, while differences between time points within the same run were analyzed via one-way ANOVA (95% significance) followed by Tukey’s HSD post hoc tests. Error bars represent standard deviations (*n* = 3). Significant differences are indicated by asterisks (*p* < 0.05 *, *p* < 0.01 **, *p* < 0.001 ***); ns = not significant.

Figure 4A shows the progression of cell growth in *C. johrii* Pisa7, measured as CDW (g L^−1^) at T0, T7, and T14 from the start of the experiment. In both replicates (Run_1 and Run_2), Tukey’s HSD post hoc test revealed a statistically significant increase in CDW during the first 168 h (T0–T7). However, in both runs, CDW stabilized over the subsequent 168 h (T7–T14), with no significant further increase. The highest CDW values were recorded at T14, with 4.90 g L^−1^ (±0.26) in Run_1 and 3.05 g L^−1^ (±0.09) in Run_2, the latter being significantly lower than Run_1.

The growth of *C. johrii* Pisa7 in FBB was also assessed by monitoring changes in BChl*a* content over time (Figure 4B). The most significant increase in BChl*a* occurred during the first 168 h (T0–T7) of inoculation for both runs, with Run_2 showing significantly higher values than Run_1 after the first 48 h (T2). Between 168 h (T7) and 336 h (T14), the BChl*a* content in Run_1 stabilized, with no significant variations. In contrast, Run_2 exhibited a statistically significant reduction in BChl*a* between 216 h (T9) and 264 h (T12), followed by a subsequent increase in the next interval. The maximum BChl*a* levels were observed at the end of the incubation period (T14), reaching 25.92 µg mL^−1^ (±2.41) and 28.98 µg mL^−1^ (±0.90) in Run_1 and Run_2, respectively, with no significant differences between the two runs.

The accumulation of PHB (% w PHB/w cells) was evaluated at T0, T7, and T14 for both runs (Figure 4C). The most substantial increase in PHB was observed during the first 168 h of inoculation (T0–T7), with increments of +11.85% w PHB/w cells (±1.03) and +9.90% w PHB/w cells (±0.93) in Run_1 and Run_2, respectively. Over the subsequent 168 h (T7–T14), no significant changes in PHB content were detected in either run. At T14 (336 h), comparing the two trials, the first experiment (Run_1) reached significantly higher PHB levels compared to Run_2 (*p* < 0.05, *), with final values of 15.17% w PHB/w cells (±0.43) and 11.51% w PHB/w cells for Run_1 and Run_2, respectively.

The PHB accumulation trends described above align with volumetric production and the hourly and daily productivities presented in Table 4. Run_1 achieved an average productivity of 744.22 ± 61.22 mg PHB L^−1^, corresponding to a daily productivity of 48.82 ± 4.34 mg PHB L^−1^ d^−1^, while Run_2 recorded lower values, with 352.01 ± 57.78 mg PHB L^−1^ and 21.33 ± 4.55 mg PHB L^−1^ d^−1^.

Figure 4D shows the temperature profiles recorded during the two experiments, which were conducted under uncontrolled room temperature conditions to minimize energy input. Starting 24 h after inoculation (T1), Run_2 showed significantly higher temperatures than Run_1. Starting 144 h after inoculation (T6), the temperatures of Run_2 stabilized, with minimum fluctuations between 30.1 °C (±0.25) and 29.4 °C (±0.31) until the end of the experiment. In contrast, the temperatures of Run_1 showed more pronounced variations during the same period, fluctuating from a maximum of 27.6 °C (±0.30) to a minimum of 24.6 °C (±0.25).

Table 5 summarizes the variation in the concentration of key compounds (carbohydrates, organic acids, ethanol, and ammonium) during the photofermentative process at different time points (T0, T7, and T14) for both experimental trials (Run_1 and Run_2).

The concentrations of maltotriose and maltose decreased significantly over time in both runs. In Run_1, maltotriose was completely consumed by T14, whereas a small residual amount of 0.02 g L^−1^ (± 0.01) remained in Run_2. A similar trend was observed for maltose, which was fully consumed by T14 in both runs. However, the reduction in maltose concentration between T0 (0 h) and T14 (168 h) was significantly greater in Run_2 compared to Run_1.

The lactate concentration decreased significantly between T0 and T7 in both runs. In contrast, no statistically significant change was observed between T7 and T14, although a slight increase was noted in Run_2 during this period. The acetate concentration, on the other hand, increased significantly over time in both runs, reaching final concentrations of 0.75 g L^−1^ (±0.03) in Run_1 and 0.97 g L^−1^ (±0.25) in Run_2 at T14. No significant differences in acetate levels were observed between the runs at any time point.

The ethanol concentration remained relatively stable between T0 and T7 in both runs. However, a significant increase was observed between T7 and T14 in Run_1, with ethanol levels reaching a peak of 1.58 g L^−1^ (±0.00). In contrast, although ethanol in Run_2 also peaked at 2.21 g L^−1^ (±0.52) during the same period, the increase between T7 and T14 was not statistically significant.

Ammonium levels remained relatively constant between T0 and T7 in both runs but showed a significant reduction by T14, reaching 15.53 mg L^−1^ (±0.32) in Run_1 and 11.60 mg L^−1^ (±2.18) in Run_2. No significant differences in ammonium concentrations were detected between the runs at any time point.

## 4. Discussion

The DF of bread waste aimed to maximize the yield of lactic acid, making the waste suitable for the subsequent PF step. Indeed, PNSB can oxidize lactic acid into pyruvate, which can be further converted into acetyl-CoA, a key precursor for PHB accumulation [41]. The DF was conducted at 37 °C by inoculating bread waste with *L. amylovorus* DSM 20532 (1 × 10^9^ CFU mL^−1^), applying the methodology described by Adessi et al. [26]. In their study, Adessi et al. [26] reported a peak lactic acid production of 4.93 ± 0.29 g L^−1^ after 18 h. In contrast, our optimization trials in 100 mL flasks yielded a maximum lactate concentration of 2.72 ± 0.04 g L^−1^ at 120 h (Figure 1), after which it declined, likely due to lactic acid-consuming microbes. These differences in yield and duration may be attributed to the higher dilution (7.5% vs. 10% *w*/*v* used by Adessi et al. [26]) in this study, as well as the inherent variability of food waste composition.

Unlike the findings of Adessi et al. [26], our study observed an unexpected production of acetic acid during DF (Figure 1). This production peaked after 120 h of incubation before declining. Acetate production cannot be attributed to *L. amylovorus* DSM 20532, which belongs to obligately homofermentative LAB [42], but could be due to the presence of other microorganisms already present in the bread wastes, which were not sterilized before use. The decision not to sterilize the bread waste reflects the aim of simulating more realistic, scalable conditions, as dark fermentation was primarily intended to enrich the substrate with organic acids, especially lactic acid, rather than to ensure strict axenic conditions.

The simultaneous production of lactate and acetate is noteworthy, as acetate can be efficiently assimilated in the metabolic pathway for acetyl-CoA synthesis, as extensively documented [43,44,45]. Once the duration of DF was optimized, FBB production was carried out in 3L Erlenmeyer flasks.

At the end of the DF process, the FBB was supplemented with digestate, obtained from an AD process, and a phosphate buffer. The addition of digestate enriches bread waste with essential macro- and micronutrients required for PNSB growth, eliminating the need for chemical supplements that would increase process costs. Indeed, during anaerobic digestion, macronutrients (N, P, K, S, Mg, Ca) and micronutrients (B, Cl, Cu, Fe, Mn, Mo, Ni, Zn) concentrate in the digestate [46].

The macro- and micronutrient composition of FBB after digestate addition (Table 2) shows higher concentrations of Ca, K, Mg, Na, and S compared to the synthetic RPN medium. However, the lower or undetectable levels of trace elements (B, Co, Cu, Fe, Mn, Mo, Ni, Zn) in FBB compared to RPN may help mitigate the adverse effects of macronutrient excess and favor PHB synthesis [47,48]. The absence of Fe, Mo, and Ni inhibits nitrogenase and hydrogenase, reducing bio-H_2_ production, which competes for reducing power with PHB synthesis, potentially increasing PHB yield [18,19]. The digestate proved optimal for supplying essential nutrients while maintaining ideal conditions for PHB production.

Scaling-up from 100 mL to 3L did not reduce acetate and lactate production efficiency (Table 3). The lactate content of the FBB substrates was higher than that of the synthetic RPN lactate medium (1.8 g L^−1^) and consistent with levels reported in other studies on PHB production by PNSB using synthetic media with lactate as a carbon source, confirming its adequacy [49,50]. Conversely, the acetate content was lower than the minimum recommended level (2.15 g L^−1^) to ensure sufficient PHB accumulation [7]. However, the low ammonium content of the FBB medium significantly enhances its C/N ratio, making it highly favorable for PHB production. In all three PF tests, the FBB C/N ratio was more than twice the minimum recommended value of 30 [7].

The preliminary screening on FBB aimed to identify the strain with the highest potential for PHB production, to be scaled up in a 5 L photobioreactor. Six different strains were tested, three belonging to *R. palustris* (42OL, AV33, CGA009), recognized for their metabolic versatility on various substrates, and three others including two species of *C. johrii* (Pisa7, 9Cis) and one of *C. sphaeroides* (F17). These latter strains were chosen for their distinct ecological origins (Table 1), which shaped their unique metabolic traits and ability to thrive under nutrient-limited conditions. All six strains had previously been assessed by Mugnai et al. [35] for their ability to produce PHB on both synthetic media and olive oil effluents.

*R. palustris* strains (42OL, AV33, CGA009) exhibited a metabolism more oriented towards biomass growth than PHB accumulation (Figure 2). Specifically, *R. palustris* 42OL and *R. palustris* CGA009 showed a significant increase in CDW over 336 h, whereas *R. palustris* AV33 exhibited an increase in CDW that was not statistically significant. In all cases, the PHB concentration did not exceed 10% w PHB/w cells at T14. These results are consistent with previous studies reporting the generally low PHB accumulation ability of *R. palustris* strains compared to other purple non-sulfur bacteria. In particular, Mugnai et al. [35] observed that *R. palustris* accumulated significantly lower amounts of PHB than both *C. sphaeroides* and *C. johrii* when cultivated in synthetic media and in agro-industrial waste-based substrates. Conversely, *Cereibacter* strains demonstrated higher PHB accumulation, with *C. johrii* Pisa7 achieved the highest value at 50.73% (±10.60) w PHB/w cells, significantly outperforming *C. sphaeroides* F17 (26.47% ± 11.58) and *C. johrii* 9Cis (22.31% ± 0.77) (Figure 2). *C. johrii* Pisa7 also exhibited the highest absolute growth, with a CDW increase of +1.26 g L^−1^ (±0.15). This dual capacity for high biomass and PHB yield makes *C. johrii* Pisa7 particularly promising for industrial PHB production.

From the TEM analysis (Figure 3), *C. johrii* Pisa7 shows a notable difference compared to the other *Cereibacter* strains. Despite its high PHB accumulation percentage, *C. johrii* Pisa7 exhibits the smallest cell and PHB inclusion sizes. The literature suggests that smaller, more abundant PHB granules can optimize intracellular space utilization, ultimately enhancing overall PHB production efficiency [51]. Based on these results, *C. johrii* was selected for scale-up in a 5 L photobioreactor, highlighting its promising potential for efficient biopolymer production on an industrial scale.

Process scale-up was conducted in a 5 L photobioreactor equipped with selected LED lights capable of emitting light in the yellow (λ = 593 nm) and near-infrared (λ = 860 nm) regions. The light intensity was increased stepwise. Initially set at 150 μmol photons m^−2^ s^−1^ to allow *C. johrii* Pisa7 to adapt to the substrate FBB, it was raised to 250 μmol photons m^−2^ s^−1^ after 24 h and to 400 μmol photons m^−2^ s^−1^ after 120 h. This adjustment ensured sufficient light penetration, preventing productivity loss caused by cell growth.

In both experiments (Run_1 and Run_2), *C. johrii* Pisa7 showed a significant increase in CDW between T0 and T7, stabilizing from T7 to T14 (Figure 4A). Run_1 had a higher CDW than Run_2 at both T7 and T14. Lactic acid consumption (Table 5) reflected this trend, with significant utilization between T0 and T7 to support growth and PHB synthesis, followed by stabilization. Growth stabilized after T7 may be due to depletion of essential macro- or micronutrients, explaining the residual lactic acid at the end of the tests.

The reactor temperature was not actively controlled, as the ambient room temperature remained stable and consistently within or close to the optimal range for PHB production (24–30 °C) [7] (Figure 4D). Despite the lower temperature in Run_1, its CDW was significantly higher than that of Run_2 at both T7 and T14 (Figure 4A). This can be attributed to a greater accumulation of PHB in Run_1 compared to Run_2. The lower initial temperatures (20–24 °C) in Run_1 may have induced metabolic stress, promoting the accumulation of storage compounds such as PHB at the expense of active cell growth. As shown in Figure 4C, PHB accumulation was higher in Run_1 than in Run_2 at both T7 and T14. However, a statistically significant difference was observed only at T14 (*p* < 0.05, *). The absence of significant differences at T7 suggests that the temperature effect becomes more pronounced over time. Indeed, during the early phase (T0–T7), PHB accumulation followed a similar trend in both conditions.

The growth of *C. johrii* Pisa7 was also assessed by monitoring BChl*a* content over time (Figure 4B), which mirrored the general growth trend. A significant increase in BChl*a* was observed in both experiments, during the first 168 h, followed by stabilization. However, no statistically significant differences in BChl*a* were observed between Run_1 and Run_2 at T7 and T14, indicating that the increase in CDW did not correlate with a proportional rise in BChl*a*. This discrepancy can be attributed to the fact that BChl*a* synthesis is primarily influenced by light intensity and quality, which was the same in both runs. Although BChl*a* content provides a reliable indication of growth tendency, it is not directly related to CDW, which is influenced by other factors, such as PHB accumulation.

The consumption of lactate (Table 5) reflects the growth of *C. johrii* Pisa7, showing a significant decrease during the first 168 h (T0–T7), followed by stabilization with no significant variations. This indicates that lactate is utilized as the main carbon source to support both growth and PHB accumulation. In contrast, ammonium (Table 5) remains stable during the first 168 h but decreases in the subsequent phase (T7–T14). The lack of ammonium consumption in the initial phase may be explained by the microorganism relying on internal nitrogen reserves accumulated during its growth on the synthetic RPN medium during inoculum preparation. Nitrogen is subsequently consumed between T7 and T14 to sustain metabolic processes related to maintenance and adaptation, even in the absence of further cell growth.

The concentrations of maltose, maltotriose, acetic acid, and ethanol (Table 5) were monitored during PF. A reduction in complex sugars (maltose and maltotriose) and a consistent increase in acetic acid and ethanol suggested possible contamination. The potential contamination observed in the photobioreactor could mainly be attributed to the fact that the system was not sterilized but only subjected to a disinfection procedure. This choice was dictated by the large size of the reactor, which made autoclave sterilization technically impractical. However, this approach better reflects industrial-scale operations, where complete sterilization is often not feasible.

At the end of the PF trials, *C. johrii* Pisa7 accumulated PHB at levels of 15.17% (±0.43) w PHB/w cells and 11.51% (±1.60) w PHB/w cells in Run_1 and Run_2, respectively. These percentages are over 70% lower than those observed during screening experiments. However, the screening experiments were conducted at a working volume of 100 mL, approximately 50 times smaller than the bioreactor volume, a difference that likely contributed to the observed loss of efficiency.

The results of this study appear promising when compared to those reported in the literature, as extensively reviewed by Sali and Mackey [41] and by Montiel-Corona and Buitrón [19]. A comparison with relevant studies is summarized in Table 6, which reports PHB production by different pure or mixed PNSB cultures, grown on dark fermented organic waste. Notably, most literature data refer to small-scale systems (≤400 mL), while the present study was conducted in a 5 L photobioreactor. Our results are in line with those reported by *Rhodopseudomonas sp*. S16-VOGS3, which was cultivated in a 4 L photobioreactor on dark fermented cheese whey, reaching 18% PHB/CDW [52]. In contrast, higher PHB contents (up to 83% PHB/CDW) were achieved with *Rhodobacter sphaeroides* AV1b grown on dark fermented municipal or food waste [53,54], but these results were obtained in 400 mL batch systems under highly controlled conditions, making them less directly comparable to this scale-up trials. Moreover, when comparing the PHB production of *C. johrii* Pisa7 cultivated in 100 mL obtained in this study during preliminary photofermentation tests, with other studies conducted at similar volumes [55,56,57], its PHB content was found to be significantly higher. This highlights the strain’s strong potential for efficient PHB accumulation. Overall, these comparisons confirm the robustness and scalability of the process developed in this work. Furthermore, no bio-H_2_ production was detected in any tested strains, either during screening experiments or in photobioreactor tests, confirming that FBB supports selective PHB production by reducing competition between metabolic pathways. This may also be due to the composition of the medium used in this study. While Adessi et al. [26] enhanced hydrogen production by adding ferric citrate and magnesium sulfate (key elements for nitrogenase activity), we only supplemented the FBB with digestate from AD and a phosphate buffer. The lack of supplementation with iron and sulfate, and their possible deficiency in the digestate, may have limited bio-H_2_ production and instead promoted PHB accumulation, highlighting the advantage of using FBB to increase PHB yield.

In the literature, most studies reporting higher PHA concentrations rely on synthetic substrates, which, while effective, are costly and less environmentally sustainable. By contrast, this study employed a real, complex substrate derived from bread waste, with minimal medium modifications, demonstrating the technical feasibility of PHB production at the larger scale and under conditions more closely aligned with potential industrial applications. However, the transition to an industrial-scale process still faces significant limitations, particularly the need for a detailed quantification of the energy inputs associated with the two-stage system, including both dark fermentation and photofermentation. It is essential to assess the energy costs related to light management, temperature control, and substrate pre-treatment, as well as those required for PHB extraction, which remains one of the most economically demanding steps. Additionally, harvesting biomass from low-density phototrophic cultures poses major efficiency and cost challenges. Further research is therefore needed to overcome these technical barriers, improve process efficiency, and validate the system’s performance over time under realistic, non-sterile conditions representative of the industrial sector.

## 5. Conclusions

This study demonstrated the feasibility of using bread waste as a substrate for PHB production via a combined DF and PF process. During DF, *L. amylovorus* DSM 20532 enriched bread waste effluent with lactate and acetate in 120 h, generating a substrate suitable for PF. The addition of digestate supplied essential macro- and micronutrients, eliminating the need for chemical additives and enhancing process sustainability.

Among the six tested PNSB strains in small-scale experiments (100 mL bottles), *C. johrii* Pisa7 showed the highest PHB accumulation and biomass increase, highlighting its potential as the most promising strain for industrial applications. Consequently, *C. johrii* Pisa7 was selected for scale-up experiments conducted in a 5 L photobioreactor equipped with LED lights tuned to the absorption peaks of PNSB bacteriochlorophylls. In these trials, *C. johrii* Pisa7 accumulated PHB up to 15.17% ± 0.43 w PHB/w cells. While reduced efficiency was observed compared to small-scale tests, PHB yields remained comparable to those reported in similar studies, validating the scalability of the process.

From an environmental perspective, further research is needed to assess the overall energy consumption of the process, including light management, temperature control, and sterilization. It is also important to explore integration with renewable energy sources. In particular, a thorough evaluation of these energy inputs through a life cycle assessment (LCA) will be essential to establish a sustainable and scalable model for the industrial valorization of agro-industrial waste into bioplastics.

In conclusion, this study contributes to advancing a circular bioeconomy by transforming waste streams into sustainable materials. Addressing technical, economic, and policy challenges will require interdisciplinary collaboration to unlock its full potential.

## Figures and Tables

**Table 1:** List of used strains and details about their origin.

Species	Origin	Reference
*Rhodopseudomonas palustris* strain 42OL	Sugar refinery waste treatment pond; Castiglion Fiorentino (AR), Italy.	[36]
*Rhodopseudomonas palustris* strain AV33	Trophic lake; Pozzuoli (NA), Italy.	[37]
*Rhodopseudomonas palustris* strain CGA009	Chloramphenicol-resistant derivative of *R. palustris* CGA001; courtesy of Prof. C. S. Harwood, University of Washington.	[38]
*Cereibacter johrii* strain 9Cis	Dairy effluent; Bologna (BO), Italy.	[35]
*Cereibacter johrii* strain Pisa7	Lake; San Rossore (PI), Italy.	[35]
*Cereibacter sphaeroides* strain F17	Sewage treatment pond; Florence (FI), Italy.	[35]

**Table 2:** Composition in metal and other inorganic elements of the FBB. Data are expressed as mean ± standard deviation (*n* = 3). nd = not detected.

Compounds	Concentration (g L^−1^)
B	nd
Ca	1.27 (±0.53)
Cd	nd
Co	nd
Cr	nd
Cu	nd
Fe	nd
K	2.74 (±0.24)
Mg	0.66 (±0.012)
Mn	nd
Mo	nd
Na	8.76 (±2.14)
Ni	nd
S	0.14 (±0.007)
Si	0.042 (±0.003)
Zn	nd

**Table 3:** Composition of substrates used in the photofermentation tests, including lactate, acetate, ammonium, the C/N ratio, and organic acids yield. Values are presented as mean ± standard deviation (*n* = 3). Different lowercase letters indicate significant differences (*p* < 0.05) according to one-way ANOVA followed by Tukey’s post hoc test. As no significant differences were found, all values within each parameter share the same letter (a).

Compounds	FBB for the Screening Test	FBB for Run_1	FBB for Run_2
Lactate (g L^−1^)	2.64 (±0.06) ^a^	3.12 (±0.30) ^a^	2.96 (±0.01) ^a^
Acetate (g L^−1^)	0.20 (±0.04) ^a^	0.19 (±0.05) ^a^	0.20 (±0.05) ^a^
Ammonium (mg L^−1^)	25.32 (±1.95) ^a^	25.66 (±2.90) ^a^	27.90 (±4.96) ^a^
C/N ratio	67.27 (±2.52) ^a^	77.50 (±6.70) ^a^	67.95 (±1.37) ^a^
Organic acids yield (%)	5.89 (±0.22) ^a^	6.91 (±0.57) ^a^	6.56 (±0.13) ^a^

**Table 4:** Volumetric PHB production at T14 (mg PHB L_cult_^−1^), PHB productivity per hour (mg PHB L_cult_^−1^ h^−1^), and PHB productivity per day (mg PHB L_cult_^−1^ d^−1^) across different strains in the screening batch experiment and experimental runs in the photobioreactor. Values are presented as mean ± standard deviation (*n* = 3). Statistical significance was assessed using one-way ANOVA (95% confidence level) followed by Tukey’s HSD post hoc test. Different lowercase letters (a, b) indicate significant differences among strains in the screening experiment, while different uppercase letters (A, B) indicate significant differences between photobioreactor runs (*p* < 0.05).

Strain/Run	mg PHB L_cult_^−1^	mg PHB L_cult_^−1^ h^−1^	mg PHB L_cult_^−1^ d^−1^
*R. palustris* 42OL	27.11 (±8.36) ^b^	0.08 (±0.02) ^b^	1.94 (±0.60) ^b^
*R. palustris* AV33	159.68 (±42.02) ^b^	0.48 (±0.13) ^b^	11.41 (±3.00) ^b^
*R. palustris* CGA009	44.66 (±17.66) ^b^	0.13 (±0.05) ^b^	3.19 (±1.26) ^b^
*C. johrii* 9Cis	66.13 (±8.51) ^b^	0.20 (±0.03) ^b^	4.72 (±0.61) ^b^
*C. johrii* Pisa7	1083.76 (±220.05) ^a^	3.23 (±0.65) ^a^	77.41 (±15.72) ^a^
*C. sphaeroides* F17	179.47 (±92.29) ^b^	0.53 (±0.27) ^b^	12.82 (±6.59) ^b^
Run_1	744.22 (±61.22) ^A^	2.03 (±0.18) ^A^	48.82 (±4.34) ^A^
Run_2	352.01 (±57.78) ^B^	0.89 (±0.19) ^B^	21.33 (±4.55) ^B^

**Table 5:** Variation in concentration of key compounds during PF, at different time points (T0, T7, T14) for Run_1 and Run_2. Values represent the mean ± standard deviation (*n* = 3). nd = not detected. Statistical differences between runs at the same time point were assessed using independent t-tests, while differences between time points within the same run were analyzed via one-way ANOVA (95% significance) followed by Tukey’s HSD post hoc tests. Statistically significant differences (*p* < 0.05) between time points within the same run are indicated by lowercase letters (a, b, c), while differences between runs at the same time point are denoted by uppercase letters (A, B).

Compound	Run	T0 (0 h)	T7 (168 h)	T14 (336 h)
Maltotriose (g L^−1^)	Run_1	0.73 (±0.17) ^a, A^	0.16 (±0.04) ^b, A^	nd
	Run_2	0.67 (±0.11) ^a, A^	0.09 (±0.05) ^b, A^	0.02 (±0.01) ^b^
Maltose (g L^−1^)	Run_1	0.28 (±0.07) ^a, A^	0.17 (±0.02) ^b, A^	nd
	Run_2	0.24 (±0.10) ^a, A^	0.04 (±0.02) ^b, B^	nd
Lactate (g L^−1^)	Run_1	3.12 (±0.30) ^a, A^	2.15 (±0.03) ^b, A^	1.98 (±0.06) ^b, A^
	Run_2	2.96 (±0.01) ^a, A^	1.99 (±0.41) ^b, A^	2.27 (±0.53) ^b, A^
Acetate (g L^−1^)	Run_1	0.19 (±0.05) ^a, A^	0.31 (±0.02) ^b, A^	0.75 (±0.03) ^c, A^
	Run_2	0.20 (±0.05) ^a, A^	0.36 (±0.09) ^b, A^	0.97 (±0.25) ^c, A^
Ethanol (g L^−1^)	Run_1	1.21 (±0.12) ^a, A^	1.24 (±0.12) ^ab, A^	1.58 (±0.00) ^b, A^
	Run_2	0.95 (±0.11) ^a, A^	1.29 (±0.28) ^ab, A^	2.21 (±0.52) ^b, A^
Ammonium (mg L^−1^)	Run_1	25.66 (±2.90) ^a, A^	25.33 (±2.65) ^a, A^	15.53 (±0.32) ^b, A^
	Run_2	27.90 (±4.96) ^a, A^	27.63 (±0.76) ^a, A^	11.60 (±2.18) ^b, A^

**Table 6:** PHB production by pure or mixed PNSB cultures grown on dark fermented organic waste. The table includes the type of inoculum and substrate, PHB content (% of CDW), volumetric PHB production (mg L^−1^), working volume (WV), light source used during photofermentation, and reference (Ref.).

Inoculum	Substrate	Light	PHB (or PHA) (% CDW)	PHB (mg L_cult_^−1^)	WV (mL)	Ref.
*Rhodopseudomonas capsulatus*	DF fruit and vegetable waste	White	24	-	76	[55]
*Rhodopseudomonas capsulatus*	DF fruit and vegetable waste	White	5	-	76	[56]
*Rhodobacter sphaeroides* AV1b	DF food waste	White	80	800	400	[53]
*Rhodobacter sphaeroides* AV1b	DF municipal organic waste	White	83	882	400	[54]
*Rhodopseudomonas* sp. S16-VOGS3	DF cheese whey	White	18	232	4000	[52]
Mixed photosynthetic culture	DF food waste	Infrared	19 (PHA)	-	100	[57]
*C. johrii* Pisa7	DF bread waste (FBB)	White	50.73	1083.76	100	This study
*C. johrii* Pisa7 (Run_1)	DF bread waste (FBB)	Selected (593 and 860 nm)	15.17	744.22	5000	This study

## Data Availability

The data presented in this study are available in the article.

## Abbreviations

The following abbreviations are used in this manuscript:

PHAs	Polyhydroxyalkanoates
PHB	Poly-β-hydroxybutyrate
Scl-PHA	Short-chain-length polyhydroxyalkanoate
PNSB	Purple non-sulfur bacteria
PF	Photofermentation
Bio-H2	Biohydrogen
DF	Dark fermentation
LAB	Lactic acid bacteria
BChls	Bacteriochlorophylls
FBB	Fermented bread broth
AD	Anaerobic digestion
HPLC	High-performance liquid chromatography
CDW	Cell dry weight
TEM	Transmission electron microscopy
OD660	Optical density at 660 nm
BChla	Bacteriochlorophyll a
